# A systematic review of COVID-19’s impact on pregnancy outcomes

**DOI:** 10.1097/MS9.0000000000003282

**Published:** 2025-04-25

**Authors:** Mojgan Mokhtari, Hamidreza Kouhpayeh

**Affiliations:** a Shahid AkbarAbadi Clinical Research Development Unit (SHACRDU), School of Medicine, Iran University of Medical Sciences, Tehran, Iran; b Infectious Disease and Tropical Medicine Research Center, Research Institute of Cellular and Molecular Sciences in Infectious Diseases, Zahedan University of Medical Sciences, Zahedan, Iran; c Department of Tropical and Infectious Diseases, Zahedan University of Medical Sciences, Zahedan, Iran

**Keywords:** COVID-19, maternal, neonatal, pregnancy, SARS-CoV-2

## Abstract

**Background:**

Contradictory data exists regarding COVID-19’s impact on pregnancy outcomes. This study will compare pandemic pregnancy outcomes to pre-pandemic levels in local, regional, and national populations.

**Methods:**

We searched three international electronic databases (PubMed) for research on COVID-19 infection and pregnancy outcomes from the first accessible to 10 December 2021. We included articles on COVID-19’s effects on pregnancy, maternal, and neonatal outcomes, using pregnancy as the main endpoint.

**Results:**

Twenty-eight studies were examined. The exposed population sampled 1 476 827, and the total sample was 23 819 822. One-third of studies found a pandemic-related increase in maternal mortality. Six of fourteen stillbirth studies showed a substantial increase. Ten of fourteen studies found no substantial increase in preterm birth rates. After the pandemic, postnatal depression, maternal anxiety, or both increased in five of nine mother mental health studies.

**Conclusion:**

There is a significant increase in postnatal maternal mental disorders and a probable increase in maternal mortality and stillbirth compared to before the pandemic. However, our study’s findings might result from healthcare inefficiency. COVID-19 vaccination is highly recommended for pregnant and breastfeeding women.

**HIGHLIGHTS**

- Covid-19 significantly impacts pregnancy outcomes and maternal health.
- Increased risk of adverse events during pregnancy observed in infected women.
- Systematic review follows PRISMA guidelines for comprehensive analysis.
- Analyzed data from multiple studies to evaluate pregnancy complications.
- Findings emphasize need for enhanced prenatal care during pandemics.

## Introduction

SARS-CoV-2 causes COVID-19. Since its first incidence in December 2019, COVID-19 has caused 175 million illnesses and 3.8 million deaths^[1]^. Over 3.8 million people died globally till 15 June 2021^[2-4]^. Global health systems are strained by the COVID-19 epidemic, both economically and socially. The COVID-19 pandemic has caused several psychological, health, economic, and health behavior changes^[5]^. This epidemic has harmed people’s life and social standing.

Pregnant women and HIV-positive persons may be more susceptible to COVID-19^[6]^. The COVID-19 pandemic has raised maternal and neonatal deaths. According to the first findings, there appears to have been a major shift in the rates of stillbirth and premature delivery during the course of the epidemic. This is the case because the pandemic has now reached its peak. There have been a number of research that have demonstrated that the number of premature births in Denmark^[7]^ and Ireland^[8]^ has dropped. On the other hand, another study has discovered that the number of preterm births in Nepal has increased. On the other hand, there has not been a significant change in the number of preterm births that have occurred in Sweden^[9]^and the United Kingdom [10]. It was also noted that the similar pattern occurred with stillbirth^[10-12]^. The COVID-19 pandemic’s negative impacts extend beyond the illness itself. There are also additional factors, such as healthcare service interruption and hospital anxiety^[13]^.

Even though these controversial facts are accessible, the effect of the COVID-19 epidemic on pregnancy outcomes is still unclear. It is the intention of this study to conduct a comprehensive analysis of past research on the results of pregnancy during the pandemic in contrast to the outcomes of pregnancy prior to the pandemic in populations that are either local, regional, or national in scope.

## Methods

This systematic review follows PRISMA principles^[14]^. There were a number of outcomes that were addressed, including maternal death, neonatal mortality, preterm birth, stillbirth, low birth weight, meager birth weight, meager birth weight, maternal depression and anxiety, gestational hypertension, and gestational diabetes. For the purpose of inclusion, observational studies, which included both cohort and cross-sectional studies, were considered eligible.

Between the initial time that the information was made available and the 10th of December 2021, three international electronic databases (PubMed) were searched. When searching across foreign databases, the following categories of phrases were utilized: COVID-19, 2019-nCoV, SARS-CoV-2, and pregnancy (Appendix I). During the search process, neither language nor time constraints were imposed on the participants. A filter for journal articles was applied to the ISI database as well as the PubMed database.

We reviewed studies on how COVID-19 infection affects pregnancy, maternal, and neonatal health. The influence that the COVID-19 infection had on the woman’s pregnancy was the primary result that we concentrated on. We did not include the research that met any of the following criteria, which we have listed below:

- Any SARS species other than SARS-CoV-2 research.
- Case studies, systematic and narrative reviews, meta-analyses, grey literature, consensus papers, editorials, and comments.
- Studies that did not include a control group.
- Research conducted on particular subsets of the population that are afflicted with an illness rather than on healthy persons.
- Text in its entirety rendered in any language other than English.
- There was no access to the complete content.

Overall, our studies were limited to studies conducted in English with full text available. Studies that included a specific disease subset rather than a control group were excluded.

Two different researchers conducted their own independent screening of the titles and abstracts of records that were retrieved from online databases. Following that, qualified full texts were examined to see whether or not they contained any data that was of interest. In the event that the data did not satisfy any of the aforementioned exclusion criteria, they were extracted and placed in the Excel sheet.

The first reviewer examined extracted data. Bibliographic data (first author’s name, publication year), study country, study population (single center, multicenter, national, regional), reported outcomes, total sample size, exposed cohort sample size, data collection period (for both the pandemic and control groups), and outcomes (statistically sign) were analyzed. A second researcher settled differences along the procedure. For each qualifying study, the New-Castle Ottawa Scale (NOS) quality evaluation scale^[15]^ was used to assess its quality. Studies suitable for the research were evaluated for quality.

The study was reported by the PRISMA (Preferred Reporting Items for Systematic Reviews and Meta-Analyses) and AMSTAR (Assessing the methodological quality of systematic reviews) guidelines.

## Results

**Figure 1.**
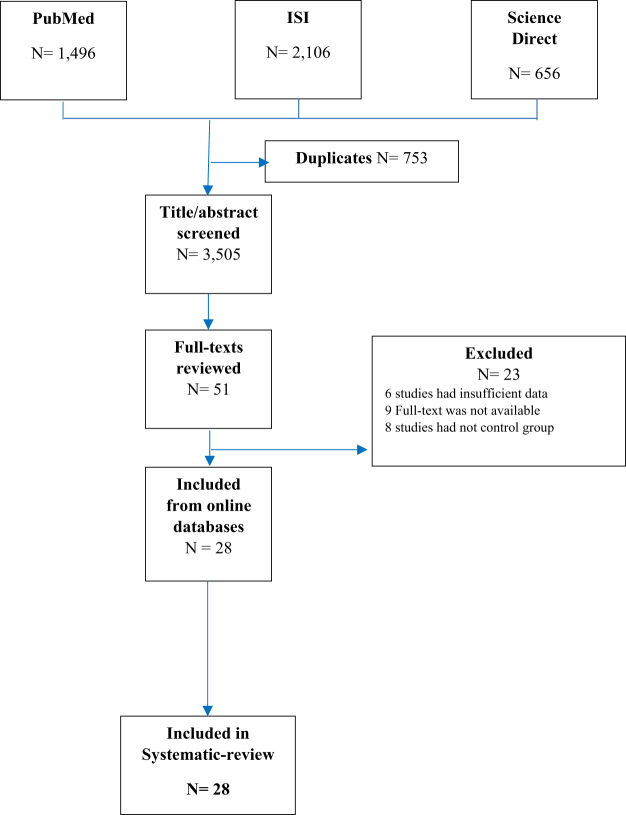
The diagrammatic representation of the selected research

The selection of studies that will be included in the current systematic review is shown in Fig. 1. Through the use of the search strategy described in methods, a total of 1496, 2106, and 656 records were discovered in the databases of PubMed, ISI, and Science Direct, respectively. Following the elimination of duplicate studies, a total of 3505 records were obtained from numerous internet databases. The inclusion criteria for the full-text review were met by 51 papers after they were screened for their titles and abstracts combined. A total of twenty-three of these full texts were disregarded for the following reasons: (1) six of the studies required further data; (2) eight of the studies did not have a control group; and (3) nine of the studies did not have the entire text available. Last but not least, our research incorporated a total of 28 studies^[10,16-42]^. The features of the selected studies, as well as the data that was retrieved from them, are presented in Table 1.

**Table 1:** Features of included research

Author, Date	Country	Study population	Reported outcome categories	Sample size of exposed cohort	Total sample size	Data collection period		Outcomes			Newcastle-Ottawa Scale score
						Pandemic group	Control group	Increased significantly during epidemic	Statistically substantial epidemic decline	Change without significance	
Ayaz, 2020^[16]^	Turkey	Single center	Maternal anxiety and depression	63	NR	12 April–27 May 2020	1 June 2018–11 April 2020	IAS II, BAI scores: moderate to severe maternal anxiety	BAI score: no maternal worry, moderate.	None	6
Berthelot, 2020^[17]^	Canada	Regional	Maternal emotions and concerns	1258	1754	2–23 April 2020	1 April 2018–1 March 2020	Negative affectivity, depression, anxiety, dissociation, and PTSD symptoms	Positive affectivity	None	6
Caniglia, 2020^[18]^	Botswana	National	Stillbirth, PTB, neonatal death	10 751	68 448	3 April–20 July 2020	Annual matched periods, 2017–19	None	PTB < 37 weeks, PTB < 32 weeks	Neonatal death, stillbirth	9
Dell’Utri, 2020^[19]^	Italy	Single center	Stillbirth	3647	9291	23 Feb–24 June 2020	23 Feb–24 June 24 2019	Stillbirth	None	None	7
Goyal, 2021 ^[22]^	India	Single center	Maternal death	633	1749	1 April–31 Aug 2020	1 Oct 2019–29 Feb 2020	None	None	Maternal death	8
Gu, 2020^[23]^	China	Single center	Gestational hypertension, gestational diabetes, PTB, stillbirth, maternal anxiety	271	582	1 Jan–29 Feb 2020	1 Jan–28 Feb 2019	Gestational hypertension, gestational diabetes	None	Stillbirth, gestational diabetes	5
Handley, 2021^[24]^	USA	Multicenter	Stillbirth, overall PTB, spontaneous PTB, iatrogenic PTB	3007	8914	1 March–30 June 2020	Annual matched periods, 2018–19	None	None	Stillbirth, PTB < 37 weeks, spontaneous PTB, iatrogenic PTB	9
Hui, 2020^[25]^	Hong Kong	Single center	Post-partum depression	954	4531	5 Jan–30 April 2020	1 Jan 2019–4 Jan 2020	Postnatal depression (EPDS score ≥10 1 day after delivery)	None	Postnatal depression (EPDS score)	5
Justman, 2020^[26]^	Israel	Single center	Gestational hypertension, gestational diabetes, PTB, stillbirth	610	1352	1 March–30 April 2020	1 March–30 April 2019	Gestational diabetes, gestational hypertension	None	Stillbirth, PTB < 37 weeks and < 32 weeks	9
Khalil, 2020^[10]^	UK	Single center	Gestational hypertension, gestational diabetes, stillbirth, PTB	1718	3399	1 Feb–14 June 2020	1 Oct 2019–31 Jan 2020	Stillbirth	Gestational hypertension	PTB < 37 weeks and < 34 weeks, gestational diabetes	7
Kumar, 2021^[29]^	India	Single center	Stillbirth	3610	9771	1 March–30 Sept 2020	1 March–30 Sept 2019	Stillbirth	None	None	9
Kumari, 2020^[30]^	India	Multicenter	Maternal death, stillbirth	3527	9736	25 March–2 June 2020	15 Jan–24 March 2020	Maternal death, stillbirth	None	None	5
Li, 2020^[31]^	China	Single center	PTB	3432	10 591	23 Jan–24 March 2020	1 Jan 2019–22 Jan 2020	PTB	None	None	9
Lumbreras-Marquez, 2020^[34]^	Mexico	National	Maternal death	1 233 491	20 806 358	1 Jan–9 Aug 2020	2011–19	No statistical analysis done	No statistical analysis done	No statistical analysis done	7
Matvienko-Sikar, 2020^[35]^	Ireland	Single center	Pregnancy-specific stress	235	445	16 June–17 July 2020	1 May 2019–29 Feb 2020	None	None	NuPDQ pregnancy stress score	5
McDonnell, 2020^[8]^	Ireland	Single center	PTB, stillbirth, neonatal death, gestational hypertension	2488	4309	1 April–31 July 2020	Annual matched periods, 2018–19	None	None	PTB < 37 weeks, PTB < 26 weeks, stillbirth, early and late infant mortality, gestational hypertension	8
Mor, 2020^[37]^	Israel	Single center	Gestational diabetes, gestational hypertension, stillbirth, PTB	1556	6120	1 Feb–30 April 2020	Annual matched periods, 2017–19	St 5 minutes, induction of labor Apgar score <7	None	Gestational diabetes, hypertension	7
Pariente, 2020^[38]^	Israel	Single center	Hypertension, diabetes, postpartum depression maternal depression, suicidal ideation	223	346	18 March–29 April 2020	1 Nov 2016–30 April 2017	None	Postpartum depression (EPDS score)	EPDS question 10: gestational hypertension, pre-eclampsia, mother suicidal thoughts positive)	5
Saleem, 2024^[43]^	India	Jammu and Kashmir, focusing on the Kashmir	COVID-19 case trends	Different	5000–18 000	1 Nov 2020–8 May 2021	Different time points during the pandemic.	Testing volumes, particularly in Srinagar.	RT-PCR positivity rate decreased from 34% in March to 28% in May.	Variations in symptomatic testing numbers between November and January remained relatively constant.	NA
			Test positivity rates (RAT and RT-PCR)								
			Case fatality rates (CFR)					RAT and RT-PCR positivity rates, peaking at 34% for RT-PCR in March.			
									RAT positivity rate showed a downward trend after initial increases.		
			Impact of healthcare and containment measures								
Silverman, 2020^[40]^	USA	Single center	Postpartum depression	155	485	12 March–12 June 2020	2 Feb–11 March 2020	None	Postnatal depression (EPDS score)	None	6
Wu, 2020^[41]^	China	Regional	Postpartum depression, maternal anxiety	1285	4124	21 Jan–9 Feb 2020	1 Jan–20 Jan 2020	EPDS for postpartum depression, EPDS-3A for maternal anxiety	None	None	9
Xie, 2021^[42]^	China	Regional	Maternal depression, maternal anxiety	689	3348	1 Jan–31 Aug 2020	1 March–31 Dec 2019	SCL-90-R score: maternal depression, anxiety	None	None	5
Du, 2021^[20]^	China	Single center	PTB, stillbirth, low birth weight	3188	7699	20 Jan–31 July 2020	20 May–30 Nov 2019	None	None	PTB <37 weeks, stillbirth, LBW	8
Gallo, 2021^[21]^	Australia	Single center	PTB	839	6899	30 March–1 May 2020	30 March–1 May 2013–2019	None	None	PTB <37, < 32 and <28 weeks	7
Kassie, 2021^[27]^	Ethiopia	Regional	Stillbirth; neonatal mortality	2476	5711	March–June, 2020	March–June, 2019	Stillbirth, Neonatal mortality	None	None	7
Kirchengas, 2021^[28]^	Austria	Single center	PTB, LBV, VLBV, ELBV	669	29 475	March–July, 2020	March–July, 2005–2019	None	None	PTB <37-32 weeks, LBW, VLBW, ELBW	7
Liu, 2021^[32]^	Canada	National	PTB, stillbirth	68 692	440 229	March–August, 2020	March–Aug, 2015–2019	PTB <37 weeks of gestation	None	Stillbirth, PTB <34, < 32 and <28 weeks	8
Llorca, 2021^[33]^	Spain	Single center	PTB, LBW	620	1589	26 May–22 Oct 2020	1 Jan–31 Aug 2018	None	LBW, PTB <34 weeks	PTB <37 weeks	7
Shah, 2021^[39]^	Canada	Regional	PTB, stillbirth	126 740	2 451 606	1 Jan–31 Dec 2020	1 July 2002–31 Dec 2019	None	PTB <32 and <28 weeks	PTB <37 weeks, stillbirth	7


PTB: premature birth, low, very low, and very low birth weights are LBV, VLBV, and ELBV.

The overall sample size of the exposed population was 1 476 827, and the total sample size was 23 819 822.

The study population of 18 studies were single-center^[10,16,19,22,23,25,26,28,29,31,33,35,37,38,40]^, two were multi-center^[24,30]^, five studies were regional^[17,27,39,41,42]^ and three studies were national^[18,32,34]^. Five studies were conducted in China, two in the United States (US), three in Israel, three in Canada, three in India, two in the United Kingdom (UK), two in Ireland, and one study in each of the following countries: Australia, Austria, Botswana, Ethiopia, Hong Kong, Italy, Mexico, Spain, and Turkey.

Observations on maternal mortality were observed in three separate research^[22,30,34]^. Only one of the three investigations, which was a multi-center analysis carried out in India, demonstrated a significant increase in maternal mortality during the pandemic in comparison to the period before the pandemic^[30]^. The results of two other studies^[22,34]^ did not show a substantial increase in the risk of maternal death; one of these studies was a statewide research project that was carried out in Mexico^[34]^.

There were fourteen studies that provided information about the number of stillbirths that occurred during and before the pandemic^[10,18-20,23,24,26,27,29,30,32,36,37,39]^. Eight studies, including two national studies, reported a non-significant increase in stillbirth^[18,20,23,24,26,32,36,39]^, and in six studies, there was a significant increase in stillbirth^[10,19,27,29,30,37]^.

Three studies included data on neonatal death^[18,27,36]^. One of the studies reported a significant increase in neonatal death compared to before the pandemic^[27]^, and two showed no significant increase in neonatal death^[18,36]^.

Reports on preterm birth were included in 14 studies^[10,18,20,21,23,24,26,28,31-33,36,37,39]^. Ten studies showed a non-significant increase in preterm birth^[10,20,21,23,24,26,28,31,36,37]^, one showed an increase^[32]^, and three showed a decrease^[18,33,39]^.

On the subject of maternal mental health, nine studies were conducted and published^[16,17,23,25,35,38,40-42]^. Five of these investigations^[16,17,25,41,42]^ reported significant increases in postnatal depression or mother anxiety after the pandemic. These studies used the GAD-7 questionnaire, Inventory of Depression and Anxiety Symptoms (Expanded Form), EPDS, Symptom Checklist 90 Revised, and Patient Health Questionnaire 9. Table 1 lists pregnancy outcomes.

Maternal mortality rates before and after the COVID-19 outbreak, based on region of residence, are shown in Table 2.

**Table 2:** Trends in maternal mortality and healthcare disruptions during COVID-19

Region	Pre-pandemic maternal mortality rate (per 100 000 live births)	During pandemic maternal mortality rate (per 100 000 live births)	% change in mortality	Antenatal care disruptions (% services affected)	Skilled birth attendance disruptions (% services affected)	Postnatal care disruptions (% services affected)
Sub-Saharan Africa^[27]^	400	460	+15%	35%	40%	38%
South Asia^[10]^	180	220	+22%	30%	25%	28%
Latin America^[34]^	70	85	+21%	20%	15%	18%
North America^[24]^	15	18	+20%	15%	10%	12%
Europe^[28]^	8	10	+25%	10%	5%	8%
East Asia^[23]^	10	12	+20%	12%	8%	10%
Middle East^[36]^	55	65	+18%	25%	20%	22%

**Figure 2.**
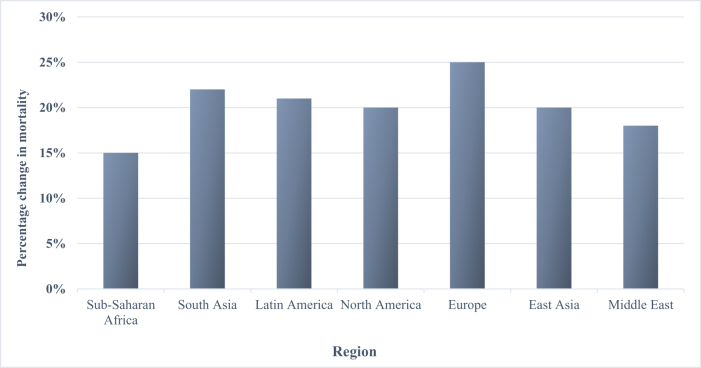
The percentage change in mortality during the coronavirus outbreak.

The percentage change in mortality during the coronavirus outbreak compared to before, based on different regions, is shown in Table 2 and Fig. 2.

## Discussion

This systematic review seeks to compile all available data on how the COVID-19 pandemic has affected pregnancy outcomes worldwide. A significant increase in the number of postnatal mothers suffering from mental illnesses, as well as a potential rise in the number of maternal deaths and stillbirths, were noticed in comparison to the time period prior to the pandemic. On the other hand, the majority of the studies did not demonstrate any discernible differences in the incidence of premature birth^[44,45]^.

Several factors have contributed to adverse pregnancy outcomes during the COVID-19 pandemic, most notably delays in accessing health services and reduced antenatal care visits. Research has shown that many pregnant women have avoided health care due to fear of exposure to the virus in health facilities^[46]^. Another study also found that restrictions imposed during the COVID-19 pandemic and reduced public transportation could have made access to necessary care more difficult^[47]^. These restrictions could have increased maternal mortality and stillbirth rates, especially in exceptional circumstances. Health service restructuring, including transferring healthcare staff to COVID-19 wards, has also reduced the capacity to provide antenatal care and put additional pressure on health systems^[48]^.

Early evidence during the pandemic suggested that the pandemic is associated with a substantial increase in pregnancy and neonatal outcomes. On the other hand, additional study suggests that the bulk of these adverse events are actually occurring in low- and middle-income nations (LMICs), whereas high-income countries (HICs) did not reveal any differences connected to these occurrences^[46]^. Resource constraints and structural challenges in LMICs compound adverse pregnancy outcomes. In addition, under-equipped medical facilities, a lack of skilled workers, and weak health infrastructure in these countries are key challenges during pregnancy^[49]^. Pregnant women in rural areas, especially in remote areas, are particularly vulnerable^[50]^. Misinformation and culturally embedded concerns have made pregnant women less willing to use the COVID-19 vaccine, which in turn has contributed to an increase in disease-related complications^[51]^. This is the case because LMICs are more likely to have their populations living in poverty. For example, a similar study in the Union Territories of India showed that COVID-19 mortality rates varied significantly due to similar reasons, including health system inefficiency and healthcare inequalities^[43]^. The latter hypothesis argues that the findings of our study and the higher risk of unfavorable pregnancy outcomes might be the result of the inefficiencies of health-care systems in dealing with the pandemic and the behaviors of the population, rather than the COVID-19 virus itself. It is also possible that the observed increase in maternal mortality is connected with decreased access to care as well as decreased attendance for routine and unscheduled pregnancy care. These findings are based on studies that were carried out in LMICs. Possible explanations for this decrease in access to care include concerns regarding the possibility of contracting COVID-19 in healthcare settings, a restriction in the availability of public transportation, or the recommendation made by the government to remain at home during lockdowns^[52]^. In HICs, the majority of prenatal care was swiftly replaced by forms of remote caregiving, such as consultations conducted over the phone or via video communication^[53,54]^. On the other hand, it is more challenging to deliver care and consultations remotely in nations with low and intermediate incomes. Due to the conditions that exist in these countries, it is possible that individuals will not receive any preventative pregnancy care^[55,56]^. The most vulnerable demographic that has the least access to health care is the one that will be most significantly affected by the COVID-19 pandemic^[57]^. This trend holds true across all settings. Even in the United Kingdom, minority ethnic groups accounted for 88 percent of all maternal deaths that occurred during the initial wave of the pandemic^[58]^. A shift in the delivery of health care and the fact that a large number of hospital staff, including maternity staff, have been deployed to support overloaded COVID-19 wards at the pandemic’s peak prevalence are two additional factors that have been linked to an increase in the number of adverse pregnancy events that have occurred during the pandemic^[46]^. Because of this latter factor, there has been a reduction in the number of staff members who are available to provide maternity care. This, in turn, has resulted in a rise in the number of adverse outcomes that are linked with pregnancy. Due to isolation, quarantine, restricted social experiences, a change in health-care delivery, extraordinary unemployment rates, financial stress, and lower psychiatric examination attendance, the pandemic has increased mental problems worldwide. Increasing maternal mental illnesses is consistent with this worldwide trend. Pregnant and postpartum women are more likely to fear COVID-19 exposure or illness for themselves, their pregnancy, or their newborns^[59]^.

Some considerations might help reduce adverse pregnancy events. Health policymakers in the ongoing pandemic must consider vulnerable individuals and communities and establish models for antenatal care that are available and feasible for this group. The importance of antenatal care should also be emphasized. National governments must consider supporting vulnerable individuals financially^[60]^. However, as Lekha *et al* have shown, public attitudes toward vaccines and misconceptions about their safety can hinder vaccination uptake among pregnant women. This is particularly the case in LMICs where misinformation, due to limited access to reliable information and cultural concerns, can lead to reduced vaccine uptake and increased adverse pregnancy-related outcomes^[61]^. A study in a regional population from India has shown that the death rate from COVID-19 can be reduced by improving access to vaccination and strengthening health services^[43]^. Women who are pregnant, nursing, trying to become pregnant, or may get pregnant should also obtain the COVID-19 vaccine. The current research suggests that having a COVID-19 vaccination during pregnancy is worth the risks^[62]^. These challenges suggest that policymakers must improve health infrastructure, raise public awareness, and provide support programs to reduce inequalities and improve pregnancy outcomes in LMICs.

One of the key strengths of the current study is that our systematic review includes a large sample size from 15 countries. The other strength is that our systematic review includes numerous maternal and neonatal outcomes, from maternal mental health to neonatal mortality. However, our study also has some limitations. As the pandemic continues and many nations face new illnesses, further qualified research may not need to be published and included in our studies.

Healthcare gaps during the pandemic require targeted strategies to reduce the harms of the disease during pregnancy. Governments and healthcare providers should prioritize the development and expansion of telehealth services. This care requires educating expectant mothers about the harms of not using the vaccine during treatment. Policymakers should also focus on providing financial and social assistance to vulnerable pregnant women, especially in LMICs. By implementing these strategies, healthcare systems can minimize disruptions to maternal care, improve pregnancy outcomes, and reduce inequalities exacerbated during pandemics.

## Conclusion

There is a significant increase in postnatal maternal mental disorders and a probable increase in maternal mortality and stillbirth compared to before the pandemic. However, our study’s findings might result from the inefficiency of healthcare systems in coping with the pandemic and population behaviors rather than the COVID-19 virus itself. Women who are pregnant or breastfeeding are strongly encouraged to get vaccinated against COVID-19. The advantages of getting vaccinated against COVID-19 much outweigh any potential risks that may be associated with immunization during pregnancy.

## Data Availability

The datasets analyzed during the current study are not applicable, as this systematic review did not generate or analyze primary data. All data referenced in this review are derived from existing literature and publicly available sources.

## Research registration unique identifying number (UIN)

Registration platform: ClinicalTrials.gov UIN: NCT06677281

## Appendix I. Search strategy and keywords used in international databases

To identify relevant studies related to COVID-19 and pregnancy, a comprehensive search was performed using the following keywords and Medical Subject Headings (MeSH terms). Boolean operators such as AND and OR were used to combine terms effectively.

**Keywords used:**

**Table:**

Category	Keywords
Virus-related terms	COVID-19, coronavirus, 2019-nCoV, SARS-CoV-2, Novel Coronavirus, Pandemic
Pregnancy-related terms	Pregnancy, pregnant women, gestation, maternal health, prenatal care, pregnancy outcome

**Search string:**

(“COVID-19” OR “2019-nCoV” OR “SARS-CoV-2” OR “novel coronavirus”) AND (“pregnancy” OR “pregnant women” OR “gestation” OR “maternal health” OR “prenatal care” OR “pregnancy outcome”)

**Databases searched:**

- PubMed
- Embase
- Scopus

Searches were limited to studies published from December 2019 to 10 December 2021, in English.
